# Heavy metal poisoning caused by Chinese folk remedies in psoriasis patients: a retrospective analysis

**DOI:** 10.1038/s41598-024-62653-4

**Published:** 2024-05-23

**Authors:** Changjiang Xue, Xuqin Du, Xiaoli Zhu, Na Wu, Qiao Ye

**Affiliations:** Department of Occupational Medicine and Toxicology, Beijing Chao-Yang Hospital, Clinical Center for Interstitial Lung Diseases, Capital Medical University, Worker’s Stadium No.8, Chao-Yang District, Beijing, 100020 China

**Keywords:** Health occupations, Risk factors, Signs and symptoms

## Abstract

Psoriasis, characterized as a chronic relapsing disease with a protracted course, often drives patients to seek relief through Chinese folk remedies (CFR). Nonetheless, the complex compositions of these remedies frequently result in unintended adverse effects, notably various types of heavy metal poisoning. Our study involved an exhaustive collection and analysis of clinical data from psoriasis patients who developed heavy metal poisoning due to CFR usage, admitted to Beijing Chao-Yang Hospital from January 2011 to October 2023. Our analysis identified 44 cases of mercury poisoning, 17 of lead poisoning, 21 of arsenic poisoning, and 4 instances of mixed heavy metal poisoning. The folk remedies used ranged from fumigation and inhalation to skin application and oral administration. Distinct pathogenic characteristics were observed in each poisoning type. After treatment with metal chelating agents, all patients experienced a reduction in heavy metal levels in their bodies, accompanied by varying degrees of symptom alleviation. This study underscores the vital necessity of opting for formal, medically approved treatments for psoriasis, thereby avoiding the hazardous consequences of unregulated folk remedies that may lead to severe heavy metal poisoning.

## Introduction

Psoriasis, a prevalent chronic inflammatory skin disease, is known for its recurring nature. Characterized by distinctive red, scaly patches on the skin, it not only affects patients’ physical appearance but also imposes a substantial psychological burden^1^. Globally, the quest for an ideal and effective treatment for psoriasis remains ongoing^2^. Although there are reports that Chinese herbal formulas, single herbs, and their active components have shown promising prospects in the clinical treatment of psoriasis^3–5^, due to the chronic and often intractable nature of the disease, many patients have not used formal traditional Chinese medicine treatments but instead resorted to Chinese folk remedies (CFR). However, these remedies often contain a complex mix of ingredients with dangerously high levels of heavy metals. Prolonged use can lead to the accumulation of these metals in the body, resulting in severe heavy metal poisoning. A lack of awareness and understanding of heavy metal poisoning among medical professionals can unfortunately lead to misdiagnosis or overlooked diagnosis of this condition. The aim of this article is to conduct a detailed review and analysis of clinical data pertaining to psoriasis patients who experienced heavy metal poisoning.

## Methods

A retrospective study of the psoriasis patients with heavy metal poisoning caused by CFR usage was conducted to provide an exhaustive report on the findings, thereby offering a comprehensive overview of the associated clinical outcomes. These patients were admitted to Beijing Chao-Yang Hospital and diagnosed and treated in accordance with Chinese occupational health standards (GBZ) during the period from January 2011 to October 2023. Patients with occupational exposure to heavy metals or lifestyle exposures other than the use of CFR were excluded. The levels of heavy metals in blood and urinary were tested for all patients. The levels of mercury and arsenic were detected using a fluorescence photometer (atomic fluorescence method), while the lead levels were detected using an atomic absorption spectrophotometer (atomic absorption method). The Research Ethics Board of Beijing Chao-Yang Hospital approved the study and waived the requirement for informed consent (LGH-2022-NO-62). All methods were performed in accordance with the ethical guidelines for human participants.

## Results

### Study population and CFR usage

Our study encompassed a total of 86 psoriasis patients suffering from heavy metal poisoning, comprising 51 males and 35 females, ranging in age from 17 to 68 years. The breakdown of cases included 44 cases of mercury poisoning, 17 of lead poisoning, 21 of arsenic poisoning, and 4 of mixed heavy metal poisoning (2 of mercury poisoning combined with lead poisoning and 2 of mercury poisoning combined with arsenic poisoning). The methods of using CFR included fumigation and inhalation (FI), skin application (SA), and oral administration (OA), as shown in Table 1. Four patients subjected their used folk remedies to heavy metal testing, revealing the following concentrations: mercury at 17.52 mg/g, mercury at 42.37 mg/g, lead at 9.81 mg/g, and a combination of mercury at 39.24 mg/g and arsenic at 22.47 mg/g.

**Table 1:** Methods and duration of CFR usage.

Methods	Mercury poisoning		Lead poisoning		Arsenic poisoning		Mixed heavy metal poisoning	
	Cases	Duration	Cases	Duration	Cases	Duration	Cases	Duration
FI	2	7 d–2 m	1	5 d	0	–	0	–
SA	6	32 d–14 m	0	–	10	7 d–2 y	0	–
OA	30	10 d–8 m	13	2 m–2.5 y	4	20 d–1 y	1	45 d
FI + OA	2	22 d–3 m	0	–	0	–	0	–
SA + OA	3	1 m–3 y	3	2 m–6 m	7	5 m–17 m	3	1 m–4 m
FI + SA + OA	1	18 d	0	–	0	–	0	–

FI, fumigation and inhalation; SA, skin application; OA, oral administration; d, day(s); m, month(s); y, year(s).

### Clinical symptoms and signs

As shown in Table 2, patients suffering from all three types of heavy metal poisoning—mercury, lead, and arsenic—presented a spectrum of clinical manifestations. These included symptoms of neurasthenia, gastrointestinal disturbances, and peripheral neuropathy. Notably, individuals with mercury poisoning frequently exhibited urinary alterations and edema. Those affected by lead poisoning predominantly experienced severe anemia and abdominal pain. Moreover, arsenic poisoning was markedly characterized by extensive dermatological damage.

**Table 2:** Clinical symptoms and signs of each type of heavy metal poisoning.

Positive symptoms and signs	Mercury poisoning	Lead poisoning	Arsenic poisoning
	Cases (%)	Cases (%)	Cases (%)
Dizziness or headache	37 (84.1)	9 (52.9)	9 (42.9)
Insomnia	29 (65.9)	6 (35.3)	8 (38.1)
Weakness	33 (75.0)	7 (41.2)	12 (57.1)
Nausea or vomiting	6 (13.6)	12 (70.6)	7 (33.3)
Decreased appetite	11 (25.0)	7 (41.2)	6 (28.6)
Memory decline	19 (43.2)	3 (17.6)	6 (28.6)
Emotional changes	9 (20.5)	2 (11.8)	3 (14.3)
Neuromuscular pain	7 (15.9)	6 (35.3)	6 (28.6)
Limb numbness	6 (13.6)	5 (29.4)	4 (19.0)
Increased foam in urine	14 (31.8)	2 (11.8)	0
Abdominal pain	2 (4.5)	11 (64.7)	3 (14.3)
Lumbago	3 (6.82)	2 (11.8)	2 (9.5)
Gingival swelling	16 (36.4)	2 (11.8)	2 (9.5)
Oral ulcer	5 (11.4)	0	1 (4.8)
Rash	3 (6.8)	0	6 (28.6)
Anemic appearance	0	5 (29.4)	1 (4.8)
Increased heart rate	5 (11.4)	1 (5.9)	4 (19.0)
Elevated blood pressure	5 (11.4)	3 (17.6)	3 (14.3)
Edema	11 (25.0)	4 (23.5)	3 (14.3)
Eye, hand or tongue tremors	8 (18.2)	2 (11.8)	1 (4.8)
Abdominal tenderness	2 (4.5)	10 (58.8)	1(4.8)
Skin pigmentation	0	0	18 (85.8)
Excessive keratinization of the skin	0	0	16 (76.2)
Skin ulcer	0	0	3 (14.3)
Finger amputation	0	0	2 (9.5)

### Auxiliary examinations

For mercury poisoning, the average blood mercury level was 0.047 mg/L (0.006–0.237 mg/L), well above the normal value of < 0.015 mg/L. Similarly, the average urinary mercury level was significantly elevated at 32.622 μmol/mol Cr (9.994–302.814 μmol/mol Cr), far exceeding the normal value of < 2.250 μmol/mol Cr. In cases of lead poisoning, the average blood lead level was observed at 521 μg/L (281–2031 μg/L), surpassing the normal upper limit of < 400 μg/L. The urinary lead levels also showed an increase, averaging at 4.891 mg/L (0.199–13.050 mg/L), which is considerably higher than the normal value of < 0.070 mg/L. For arsenic poisoning, the average blood arsenic level was found to be 72.6 μg/L (5.6–119.8 μg/L), within a broad normal range of 2.5–190.0 μg/L. The average urinary arsenic was also elevated at 1.288 mg/L (0.159–4.767 mg/L), exceeding the normal value of < 0.100 mg/L.

In 7 cases of mercury poisoning, there was a significant increase in liver enzymes. Alanine Aminotransferase (ALT) levels varied between 57 and 335 U/L, substantially higher than the normal range of 9–50 U/L. Aspartate Aminotransferase (AST) levels also showed a rise, ranging from 42 to 273 U/L, which is above the normal value of 15–40 U/L. Additionally, 2 cases exhibited elevated serum creatinine levels, measuring 115.4 mmol/L and 176.1 mmol/L, respectively. Among 5 cases of lead poisoning, ALT levels ranged from 53 to 406 U/L, and AST levels were found to be between 42 and 299 U/L. In 3 cases of arsenic poisoning, ALT varied from 48 to 133 U/L, and AST ranged from 42 to 126 U/L.

Electromyography in 3 cases of mercury poisoning, 4 cases of lead poisoning, and 1 case of arsenic poisoning showed evidence of conduction nerve damage. Additional findings included proteinuria in 17 cases of mercury poisoning, with 9 cases progressing to nephrotic syndrome characterized by significant proteinuria (3462–5085 mg/days), marked hypoalbuminemia (11.2–26.6 g/L), and hyperlipidemia. Kidney biopsy in 6 cases indicated membranous nephropathy. In lead poisoning, 14 cases showed reduced hemoglobin levels (70–111 g/L), and blood cell analysis in 6 cases revealed basophilic stippling of red cells. Skin pathology in 2 cases of arsenic poisoning was consistent with basal cell carcinoma of the skin.

### Treatment and prognosis

For patients with mercury or arsenic poisoning, sodium 2,3-dimercapto-1-propanesulfonate (DMPS) was used for chelation therapy, administered as a 250 mg intramuscular injection once daily. For lead poisoning, calcium disodium edetate (EDTA) chelation therapy was employed, with a 1 g intravenous drip once daily. The chelation therapy regimen involved administering the medication for 3 days, followed by a 4-day break, constituting one treatment course. For patients with both mercury and lead poisoning, both DMPS and calcium disodium EDTA were administered simultaneously. 24-h urinary heavy metal levels were monitored every day during the medication in each course. After several treatment courses, a significant reduction in the patients' body heavy metal content was observed, as shown in Table 3. Except for the dermatological damage in arsenic poisoning patients, which showed no significant improvement, all other symptoms and signs improved or even disappeared.

**Table 3:** 24-h urinary heavy metal levels in all patients during chelation therapy.

Treatment courses	Mercury poisoning		Lead poisoning		Arsenic poisoning	
	Cases	Urinary mercury (μg/day)	Cases	Urinary lead (mg/L)	Cases	Urinary arsenic (mg/L)
1	48	139.4–8686.9	19	0.357–4.545	23	0. 278–5.981
2	37	77.2–2962.5	18	0.333–3.668	16	0.222–1.554
3	18	66.7–828.1	11	0.199–1.786	8	0.173–0.827
4	11	52.8–336.4	9	0.174–1.525	3	0.166–0.545
5	5	47.3–201.5	6	0.172–1.228	1	0.107–0.224
6	2	50.2–164.7	3	0.993–1.004	1	0.062–0.102
7	1	16.3–38.9	2	0.726–0.995		
8			2	0.483–0.801		
9			1	0.206–0.387		
10			1	0.084–0.229		
11			1	0.023–0.068		

The normal value of urinary mercury in treatment: < 45.0 μg/day.

## Discussion

Patients with psoriasis, owing to the persistent characteristics of their ailment, frequently turn to CFR that contain significant levels of heavy metals like mercury, lead, and arsenic^6–8^. The concentration of these heavy metals in Traditional Chinese Medicine is regulated by specific ISO standards (ISO 18664-2015, GB 2762-2017). However, in the cases of four patients highlighted in this article, the levels of heavy metals in their remedies were found to surpass these established standard limits. The application methods for folk remedies primarily included oral administration, followed by fumigation and inhalation and skin application, with some patients even using multiple methods simultaneously. Although the symptoms of psoriasis lesions showed slight improvement in the short term after using these remedies, the underlying mechanism remains unclear. However, long-term use of these remedies can lead to the accumulation of heavy metals in the body, subsequently manifesting symptoms of heavy metal poisoning^9^.

Heavy metals and their compounds distribute throughout various organs and tissues of the body, where they exhibit a pronounced affinity for sulfhydryl groups. This leads to changes in the activity of sulfhydryl-based enzymes and sulfhydryl-containing receptors, resulting in damage to target organs. These damages present as specific clinical symptoms and signs of poisoning. Different heavy metals exhibit distinct clinical manifestations, and there is individual variability in patients’ toxic responses to these substances. The clinical characteristics of patients with mixed poisoning are a combination of symptoms from multiple heavy metal toxicities.

The significant neurasthenic syndrome symptoms resulting from lead, mercury, and arsenic poisoning are noteworthy. Patients commonly display symptoms like dizziness, headache, insomnia, fatigue, and memory decline, accompanied by anxiety and restlessness^10^. Gastrointestinal symptoms primarily include nausea, vomiting, a peculiar taste in the mouth, mucosal erosion, and ulceration. Additional symptoms may encompass abdominal pain, diarrhea, and abnormal liver function^11^. Neurological damage due to these toxic substances often presents as tremors, sensory abnormalities in the limbs, and movement disorders. In more severe cases, patients may suffer from toxic encephalopathy^12^.

Notably, severe mercury poisoning significantly impacts kidney function, ranging from proteinuria in milder cases to nephrotic syndrome and elevated serum creatinine levels in severe instances^13^. Among 44 patients with mercury poisoning, 9 showed pronounced lower limb edema and significant proteinuria, suggesting typical nephrotic syndrome^14^. Pathological examinations in these cases revealed membranous nephropathy^15^. Most patients with lead poisoning experience varying degrees of anemia and abdominal pain. These symptoms are often misdiagnosed as acute gastroenteritis, intestinal obstruction, or appendicitis^16^. In some cases, patients undergo exploratory laparotomy to determine the cause of their symptoms, leading to considerable physical harm and financial burden for both the patients and their families. Chronic arsenic accumulation in the skin leads to distinct skin pigmentation and hyperkeratosis in affected individuals^17^. In severe cases of arsenic poisoning, skin ulcers and even in-situ skin cancer can develop^18^.

The cases discussed in this article demonstrated that all patients showed significant reductions in urinary heavy metal levels and marked clinical improvement after several courses of treatment^19^. However, as lead tends to accumulate more in deeper tissues such as muscles and bones, a longer course of treatment is often required to reduce urinary lead levels to normal. Additionally, it was observed that even after normalizing urinary arsenic levels, the skin pigmentation and hyperkeratosis in patients with arsenic poisoning did not show significant improvement. The skin damage caused by arsenic poisoning, similar to psoriasis, requires long-term and regular treatment in dermatology for improvement.

Based on the discussion, it is recommended that psoriasis patients who have been using CFR for extended periods should undergo comprehensive heavy metal testing. This approach is crucial to avoid missing a diagnosis of heavy metal poisoning, which could delay appropriate treatment. Early detection of heavy metal poisoning allows for the immediate cessation of exposure to the toxic source and the initiation of chelation therapy, which generally leads to a favorable prognosis. The issue of heavy metal poisoning caused by CFR warrants societal attention. Patients with psoriasis should be warned against impulsive and indiscriminate medical treatments to avoid the physical harm associated with heavy metal poisoning.

Some limitations of the present study should be mentioned. First, the study is retrospective, and heavy metals poisoning caused by CFR usage may involve a wider variety of toxins and more toxic features that have not been identified. In addition, due to the limited number of cases of various types of heavy metal poisoning, more data is needed for better scientific research.

## Data Availability

The datasets used during the current study are available from the corresponding author on reasonable request.
